# ﻿Typification of names of *Weinmannia* (Cunoniaceae) of species described by José Cuatrecasas from Colombian collections

**DOI:** 10.3897/phytokeys.254.135362

**Published:** 2025-03-26

**Authors:** Carmen Ulloa Ulloa, Andrea Chaspuengal-Morales, Francisco Fajardo-Gutiérrez, Nora H. Oleas

**Affiliations:** 1 Missouri Botanical Garden, 4344 Shaw Blvd., St. Louis, Missouri 63110, USA Missouri Botanical Garden St. Louis United States of America; 2 Centro de Investigación de la Biodiversidad y Cambio Climático (BioCamb), Facultad de Ciencias de Medio Ambiente, Universidad Tecnológica Indoamérica, Machala y Sabanilla, EC170301 Quito, Ecuador Universidad Tecnológica Indoamérica Quito Ecuador; 3 Botanischer Garten Berlin, Freie Universität Berlin, Königin-Luise-Straße 6-8, 14195 Berlin, Germany Freie Universität Berlin Berlin Germany

**Keywords:** Andes, Colombia, Cuatrecasas, lectotypes, nomenclature, South America

## Abstract

We propose 18 new lectotype designations for names of *Weinmannia*, five of which are second-step typifications involving four inadvertent first-step lectotype designations, and 13 of them are designations of one single duplicate at the herbarium where deposited. José Cuatrecasas described all, but one, of the taxa from collections made in Colombia. We clarify the lectotypification for *Weinmanniaparvifoliolata* Cuatrec. Finally, we propose one lectotype designation for *W.cochensis* described by Georg Hieronymus.

## ﻿Introduction

The genus *Weinmannia* L. is the largest in the family Cunoniaceae and consists of an estimated 90 species mostly found in the Americas with two species on the Mascarene Islands (Harling 1999; Pillon et al. 2021). Bernardi (1961, 1963) was the latest author to review *Weinmannia* worldwide. A modern comprehensive taxonomic monograph for *Weinmannia* is lacking. In the last three decades, regional or country level synopses or checklists have been published for Latin America: Bolivia (Harling and Fuentes 2014), Brazil (Santos-Silva et al. 2020), Colombia (Bernal 2016; Fajardo-Gutiérrez et al. 2020), Ecuador (Bradford 1999; Harling 1999), Peru (Zarucchi 1993), Venezuela (Hokche et al. 2008; Bradford and Berry 1998), and the Southern Cone (Hopkins 2008); as well for Mexico and Central America (Morales 2010), and the Caribbean (Acevedo-Rodríguez and Strong 2012); and a summary checklist for the Western Hemisphere by Ulloa Ulloa et al. (2017).

*Weinmannia* is a genus with extremely variable morphological characters and ongoing phylogenetic studies do not support Bernardi’s sections based on leaf morphology. Neither are Bradford’s (2002) sections supported when based on inflorescence characteristics (Fajardo-Gutiérrez in preparation; Pillon et al. 2021). Moreover, *Weinmannia* seems to be an assemblage of various species complexes and possible hybrids making species delimitations difficult.

During an ongoing synoptical study of the Ecuadorian species of *Weinmannia*, as part of a larger project investigating the genus for Latin America, we realized that most species’ names described by José Cuatrecasas from Colombia need typification. As part of his studies on Andean plants, Cuatrecasas (1933, 1940, 1941, 1942, 1948 [1949]) described 37 species and varieties of *Weinmannia* from Colombia, most of them based on his collections. He usually cited his gatherings as “Typus” or “Type”, without specifying how many duplicates he studied, or in which herbaria he deposited his collections; in other words, his new taxa were based on syntypes (Turland et al. 2018: Art 9.6). Duplicates of Cuatrecasas’ collections were distributed to various herbaria in the Americas and Europe. In his review of the American species of *Weinmannia*, Bernardi (1961, 1963) cited some of those type collections in specific herbaria, but in most cases we found that a second-step lectotypification was required (Turland et al. 2018: Art. 9 Ex. 14). Harling (1999) in his treatment for the *Flora of Ecuador* series provided typification of names applied to species of *Weinmannia* occurring in Ecuador. The establishment of nomenclatural types is crucial for any discussion of taxonomic problems such as species delimitation, character evolution reconstruction, hybridization, and to improve field and herbarium identifications. In this contribution, we propose lectotypification of 14 species and four varietal names in order to fix the application of those names in anticipation of the publication of future taxonomic studies; we also clarify one previous lectotypification.

## ﻿Material and methods

We assessed the names first by reviewing the protologues and studying most of the type material deposited in the following herbaria: B, COL, F, MA, MO, P, (acronyms according to Thiers 2024). We also verified all the type images in JSTOR Global Plants (plants.jstor.org), and websites at G, P, US, and W. The names are arranged in alphabetical order of their basionyms. For each we provide the most recent accepted species name given by Bernal (2016) in the catalogue of plants and lichens of Colombia. José Cuatrecasas Arumí (1903 Camprodon, Spain-1996 Washington D.C.) was an important botanist working on the flora of tropical America. In the 1930s, Cuatrecasas worked at the Real Jardín Botánico in Madrid; from 1939 to 1947, he was associated with academic institutions in Colombia; from 1947 to 1955 with the Field Museum in Chicago. He continued his prolific career at the United States National Herbarium in Washington D.C. (Robinson et al. 1996). For this reason, we decided to designate most lectotypes in COL or F herbaria.

At the end, we include one typification of a species name described by Georg Hieronymus from Colombian collections. References to Article numbers and examples (“Art.”) in the following are according to the International Code of Nomenclature for algae, fungi, and plants (Shenzhen Code) by Turland et al. (2018).

## ﻿Nomenclatural treatment

The following specific and varietal names proposed by Cuatrecasas between 1933 and 1948 have not been typified until now. We also clarify the lectotype designation of *Weinmanniaparvifoliolata* Cuatrec.

### ﻿ Weinmannia balbisiana Kunth var. subovata

Taxon classificationPlantaeOxalidalesCunoniaceae

Cuatrec., Lloydia 11(3): 201. 1948 [1949].

32E9A6AF-6A1E-57F3-8C4D-9C79F476039F

#### Type.

Colombia • Comisaría del Caquetá, Cordillera Oriental, Quebrada del rio Hacha, abajo de Gabinete, 2100–2250 m. alt. 23-III-40, *J. Cuatrecasas 8562* (lectotype, designated here: F [barcode F0063208F image!]; isolectotypes, COL [barcode COL000001488!]), F [barcode F0063209F image!], F [barcode F0063210F image!].

#### Note.

Cuatrecasas (1948 [1949]) cited the single collection *J. Cuatrecasas 8562* housed at F as the type; however, in the herbarium F there are three duplicates of *J. Cuatrecasas 8562*; these three specimens are syntypes so we chose one of them for the lectotypification (cf., Art. 40 Ex. 3). This variety is a synonym of *W.balbisiana* Kunth according to Bernal (2016: 1126).

### ﻿ Weinmannia bogotensis

Taxon classificationPlantaeOxalidalesCunoniaceae

Cuatrec., Ciencia (México) 1: 253. 1940.

96B1B25E-1E06-532B-AD98-AAC620769248

≡ Weinmanniaauriculatavar.bogotensis (Cuatrec.) Bernardi, Candollea 18: 325. 1963.

#### Type.

Colombia • Dep. Cundinamarca, vertiente oriental de la Sierra de Bogotá en la quebrada de los Santos, 3000 m, 28-I-1940, *J. Cuatrecasas 8005* (lectotype, first-step designated by Bernardi (1963: 325) as type, second-step lectotype designated here F [barcode F0063212F image!]; isolectotypes, BC [barcode BC623896 image!], COL [barcode COL000001489!], COL [barcode COL000001490!], F [barcode F0063213F image!], P [barcode P00697246!], U [barcode U0001478 image!], US [barcode US00097243 image!]).

#### Note.

Cuatrecasas (1940) did mention the collection *J. Cuatrecasas 8005* as the type, but he did not cite the herbarium housing the type. Subsequently, Bernardi (l.c.) cited *J. Cuatrecasas 8005* housed at F as the type; however, the F herbarium has two duplicates of the preceding collection. It is construed here that Bernardi inadvertently did the first-step lectotypification process, and we herewith select one duplicate for the second-step lectotypification. This species is a synonym of *W.auriculata* D. Don in Bernal (2016: 1126), and it is likely a hybrid-origin taxon (Fajardo-Gutiérrez et al. 2020; Miranda and Fuentes 2012).

### ﻿ Weinmannia caquetana

Taxon classificationPlantaeOxalidalesCunoniaceae

Cuatrec., Caldasia 2: 19, fig. 7. 1941.

B0C7D711-88E8-556D-963D-EA2CFEDCC359

≡ W.subsessiliflorasubsp.caquetana (Cuatrec.) Bernardi, Candollea 17: 144. 1961.

#### Type.

Colombia • Comisaría del Caquetá, Quebrada del Río Hacha, 2100-2250 m. alt., 23-III-1940, *J. Cuatrecasas 8561* (lectotype, first-step designated by Bernardi (1961: 144) as type, second-step lectotype designated here, F [barcode F0063216F image!]; isolectotypes, COL [barcode COL000001512!], F [barcode F0063215F image!], US [barcode US00097250 image!]).

#### Note.

For the type, Cuatrecasas (1941) cited three collections, viz., “23-III-1940, *J. Cuatrecasas* no. 8561”, “22-III-40, *J. Cuatrecasas*, *8599-A*”, and “26-III-1940, *J. Cuatrecasas*, *8714*”. Bernardi (1961: 144) cited *J. Cuatrecasas 8561* as the type housed in the F Herbarium. We found two duplicates of this collection in the F herbarium and chose one of them for the second-step lectotypification. Bernal (2016: 1128) treats this species as a synonym of *W.subsessiliflora* Ruiz & Pav.

### ﻿ Weinmannia crenata C. Presl var. caliana

Taxon classificationPlantaeOxalidalesCunoniaceae

Cuatrec., Ciencia (México). 1: 254. 1940.

6BE7D9D4-5CEF-581B-ABAE-3463E87486ED

≡ W.sorbifoliaKunthvar.caliana (Cuatrec) Cuatrec., Lloydia 11(3): 204. 1948 [1949].

#### Type.

Colombia • Dep. El Valle, Hoya del río Cali, Bosques “El Recuerdo” 1800 m. [s.d.], *J. M. Duque-Jaramillo 1555* (lectotype, designated here, US [barcode 00097255 image!]; isolectotypes, VALLE [barcode VALLE000201 image!], VALLE [barcode VALLE000200 image!]).

#### Note.

In the protologue, Cuatrecasas (1940) did not mention a number or a herbarium for the J.M. Duque J. [José María Duque Jaramillo] collection. However, when Cuatrecasas (1948 [1949]) made the new combination in *W.sorbifolia* Kunth, he did mention ‘*Duque 1555*’ housed in the US herbarium, but without designating it as the type. We chose this duplicate as the lectotype. This variety is a synonym of *W.sorbifolia* Kunth in Bernal (2016: 1128).

### ﻿ Weinmannia cundinamarcensis

Taxon classificationPlantaeOxalidalesCunoniaceae

Cuatrec., Revista Acad. Colomb. Ci. Exact. 5: 32, fig. 18. 1942.

7326A3FE-7B09-5261-B94B-75B9A0CE04F9

#### Type.

Colombia • Cordillera Oriental; Departamento Cundinamarca; extremo sudeste de la Sabana de Bogotá, en San Miguel, bosque a 2800-3000 m. alt., colect 10 sept. 1941, *J. Cuatrecasas & R. Jaramillo 12033* (lectotype, first step designated by Bernardi (1961: 142) as type, second-step lectotype designated here: F (barcode F0063222F image!); isolectotypes, F (barcode F0063221F image!), F (barcode F0063223F image!), COL (barcode COL0000014932!), COL (barcode COL000001493!), COL (barcode COL000001494!), GH (barcode GH00043352 image!), P (barcode P02441813 image!), US (barcode US00097252 image!).

#### Note.

In the protologue, Cuatrecasas (1942) cited a single gathering as the type (“*Typus*: … 10 sept. 1941, *J. Cuatrecasas & R. Jaramillo 12033*.”), but he did not mention an institution where the type was deposited. Bernardi (1961: 142) mentioned an F duplicate as type (“…. *Cuatrecasas 12033* (typus F,”)). We, however, found three duplicates of that collection in the F herbarium, and therefore is construed here that Bernardi inadvertently did the first-step lectotypification process, and we herewith select one duplicate for the second-step lectotypification. *Weinmanniacundinamarcensis* is known only from the type locality and is accepted by Bernal (2016: 1126). However, due to intermediate characters, Fajardo-Gutiérrez et al. (2020) suggested this taxon likely is of hybrid-origin.

### ﻿ Weinmannia duquei

Taxon classificationPlantaeOxalidalesCunoniaceae

Cuatrec., Caldasia 2: 25, figs. 10E, 14. 1941.

26161DA4-78D6-5514-BC4C-8D41F378E0CB

#### Type.

Colombia • Departamento del Valle; Hoya del río Cali, “El Recuerdo”, 2500 m. alt., *J.M. Duque-Jaramillo s.n.* (lectotype, designated here: COL [barcode COL000001495!]; isolectotype (fragment) G [barcode G00357679 image!]).

#### Note.

Cuatrecasas (1941) cited a single collection as the type but did not mention the herbarium housing the type (“*Typus*: … legit *Duque-Jaramillo* [s.n.; s.d.].”). Therefore, we designate the specimen housed at the COL herbarium as the lectotype. *Weinmanniaduquei* was considered a synonym of *W.chryseis* Diels (a species described from Peru) in Bernal (2016: 1126).

### ﻿ Weinmannia magnifolia

Taxon classificationPlantaeOxalidalesCunoniaceae

Cuatrec., Caldasia 2: 25, figs. 10D, 13. 1941.

4826B523-43A7-5461-B9F9-0384BCB2A91E

#### Type.

Colombia • Dep. de Huila, entre Gabinete y Andalucía, 2300-2200 m. alt., 24-III-1940, *J. Cuatrecasas 8590* (lectotype, designated here: COL [barcode COL000001498!]; isolectotypes, COL [barcode COL000001499!], F [barcode F0063243F image!], F [barcode F0063244F image!], P [barcode P00697262!], U [barcode U0082345 image!], US [barcode US00097274 image!], US [barcode US00097275 image!]).

#### Note.

Cuatrecasas (1941) cited a single collection as the type but did not mention the herbarium housing the type (“*Typus*: … 24-III-1940, *J. Cuatrecasas*, *8590.*”). Bernal (2016: 1127) listed the “isotype” as (“IT: COL, F”). Since there was no holotype, there was no isotype. Although Bernal’s usage of the type term “isotype” is correctable to lectotype (see Art. 9.10) his citation of two herbaria and the lack of usage of the phrase “designated here” (or its equivalent) do not constitute any inadvertent lectotypification (see Art. 7.11). Furthermore, at COL, we found two duplicates of that collection, and we chose one of them, which agrees well with the protologue, for the lectotypification. This species was accepted in Bernal (2016: 1127).

### ﻿ Weinmannia myrtifolia

Taxon classificationPlantaeOxalidalesCunoniaceae

Cuatrec., Caldasia 1(2): 16, figs. 4, 5B. 1941.

95603D84-9C77-5733-9AD1-C25FCCCC975C

#### Type.

Colombia • Cordillera Oriental, vert. oriental, Dep. de Cundinamarca, Bosque en Juiquín, Quebrada Amarilla, bajo el páramo de Guasca, 2840 m. alt., 2-VI-1940, *J. Cuatrecasas 9451* (lectotype, designated here, COL [barcode COL000001501!]; isolectotypes, F [barcode F0063252F image!], F [barcode F0063253F image!], P [barcode P02441812!], U [barcode U0082346 image!], US [barcode US00097280 image!], US [barcode US00097281 image!], US [barcode US00997637 image!]).

#### Note.

Cuatrecasas (1941) cited a single collection as the type but did not mention the herbarium housing the type (“*Typus*: … 2-VI-1940, *J. Cuatrecasas*, *9451*.”). He also cited a paratype collection as “*Ya en prensa este trabajo, el Dr. A. Dugand ha recolectado en el mismo Páramo de Guasca y en lugar próximo al del tipo unos ejemplares (Dugand 2969: Cordillera oriental: Páramo de Guasca; vertiente oriental, 3100 metros alt., Julio 20, 1941)*”. Bernal (2016: 1126-1127) merely mentioned that the type was housed in COL, F, U, and US herbaria and treated this species as a synonym of *W.karsteniana* Szyszył. We selected the duplicate deposited at COL as the lectotype.

### ﻿ Weinmannia ovalis Ruiz & Pav. var. petiolata

Taxon classificationPlantaeOxalidalesCunoniaceae

Cuatrec., Lloydia 11(3): 203. 1948 [1949].

99DA847E-5066-56AE-B9E8-6E4A32E83FC5

#### Type.

Colombia • Dep. del Valle, Cordillera Occidental, Los Farallones, Quebrada de Las Nieves, lomas parameras sobre la mina El Diamante 3100–3120 m, 31-VII-1946, *J. Cuatrecasas 21820* (lectotype, designated here: F [barcode V0334900F image!]; isolectotypes, F [barcode V0334901F image!], COL [barcode COL000076221!], P [P05518589 image!]).

#### Note.

Cuatrecasas (1948 [1949]) did cite the collection *J. Cuatrecasas 21820* housed at the F Herbarium as the type; however, in the F Herbarium there are two duplicates of the collection, and both specimens lack the holotype annotation, and thus constitute syntypes; we chose one of them for the lectotypification (cf. Art. 40 Ex. 3). This variety is a synonym of *W.elliptica* Kunth according to Bernal (2016: 1126).

### ﻿ Weinmannia parvifoliolata

Taxon classificationPlantaeOxalidalesCunoniaceae

Cuatrec., Caldasia 2: 21, figs. 9, 10A. 1941.

9242036B-C6B1-541C-B936-4DA6DA3908A0

#### Type.

Colombia • Cordillera Oriental de Colombia, en el filo divisorio entre la Comisaría del Caquetá y Departamento del Huila, 2300–2400 m. alt., 22-III-1940, *J. Cuatrecasas 8486-A* (lectotype, designated by Bernardi (1961: 147): F [barcode F0063261F image!], isolectotypes: COL [barcode COL000001502!]; US [barcode US00097286 image!], US [barcode US00997638 image!]).

#### Note.

Although Cuatrecasas (1941) cited a single collection as the type, he did not mention the herbarium housing the type (“*Typus*: … 22-III-1940, *J. Cuatrecasas*, *8496*-*A.*”). Bernardi (1961: 147) assumed that the holotype was at the COL Herbarium and mentioned the duplicate at the F Herbarium as an “isotype”. Since there was no holotype, there was no isotype, nonetheless, Bernardi’s usage of the type term “isotype” is correctable to lectotype (see Art. 9.10). Therefore, Bernardi’s citation of “isotype” is treated here as the inadvertent lectotype designation; Bernal (2016: 1127) merely mentioned the isotype (“IT: COL, F”) and accepted this species.

### ﻿ Weinmannia penicillata

Taxon classificationPlantaeOxalidalesCunoniaceae

Cuatrec., Caldasia 2: 13, figs. 1, 2. 1941.

BD82DE71-E1F8-5E08-BFD7-0FB7A2F41C0F

≡ W.ovalisvar.penicillata (Cuatrec.) Cuatrec., Lloydia 11(3): 203. 1948 [1949].

#### Type.

Colombia • Cordillera Oriental sobre el filo divisorio entre el Dept. del Huila y la Comisaría del Caquetá, en Gabinete, 2300-2450 m alt., 21-III-1940, *J. Cuatrecasas 8430* (lectotype, designated here, F [barcode F0063264F image!]; isolectotypes, CM [barcode CM0754 image!], COL [barcode COL000001503!], F [barcode F0063263F image!], G [barcode G00357648 (fragment) image!], US [barcode US00097287 image!]).

#### Note.

Cuatrecasas (1941) cited a single collection as the type but he did not mention the herbarium housing it (“*Typus*: … 21-III-1940, *J. Cuatrecasas*, *8430*.”). Subsequently, he mentioned (Cuatrecasas 1948 [1949]) that the type was housed in the F Herbarium. We, however, found two duplicates of that collection at F. Therefore, it is interpreted that Cuatrecasas (1948 [1949]) inadvertently did the first-step lectotypification process, and we herewith do the second-step lectotypification process. Bernal (2016: 1126) treated this species as a synonym of *W.elliptica* Kunth.

### ﻿ Weinmannia queremalensis

Taxon classificationPlantaeOxalidalesCunoniaceae

Cuatrec., Lloydia 11(3): 193. 1948 [1949].

CEBB72C9-B167-5279-8274-1943F0673F39

#### Type.

Colombia • Departamento del Valle, Cordillera Occidental, vertiente occidental, Hoya del río Digua, lado izquierdo del río San Juan en la region de Querermal, 1540–1650 met. alt., Quebradita del Km. 51, 25-II-1947 colect. *J. Cuatrecasas 23723* (lectotype, first-step designated by Bernardi (1963: 310) as type, second-step lectotype designated here, F [barcode F0063272F image!]; isolectotypes, BC [barcode BC623891 image!], COL [barcode COL000001504!], COL [barcode COL000001506!], COL [barcode COL000001499!], F [barcode F0063273F image!], P [barcode P00697257 image!], U [barcode U0001482 image!], US [barcode US00097295 image!], US [barcode US00097296 image!], VALLE (not seen).

#### Note.

Within the protologue, Cuatrecasas (1948 [1949]) did cite a single collection as the type (“*Type*: … 25-II-1947 colect. J. Cuatrecasas 23723.”); he however, mentioned three herbaria (F, VALLE, US) as housing the type, and he thus did not indicate the holotype and only cited syntypes. Bernardi (1963: 310) mentioned *Cuatrecasas 23723* (“F; typus *W.queremalensis*”); we, however, found two duplicates of this collection in that herbarium. Bernardi inadvertently did the first-step lectotypification process, and we herewith select one duplicate for the second-step lectotypification. *Weinmanniaqueremalensis* is considered a synonym of *W.latifolia* C. Presl. in Bernal (2016: 1127), who cited the type as housed in COL, F, US, and VALLE herbaria.

### ﻿ Weinmannia sclerophylla

Taxon classificationPlantaeOxalidalesCunoniaceae

Cuatrec., Caldasia 2: 17, figs. 5C, 6. 1941.

9C5727EA-F994-5692-B467-C41B9B2BDAC0

≡ W.sorbifoliaKunthvar.sclerophylla (Cuatrec.) Cuatrec., Lloydia 11(3): 204. 1948 [1949].

#### Type.

Colombia • Cordillera Oriental de Colombia, vertiente occidental, Dep. del Huila, entre Gabinete y Andalucía, bosque 2300-2200 m. alt., 25-III-1940, *J. Cuatrecasas 8691* (lectotype, designated here, COL [barcode COL000001518!]; isolectotypes, F [barcode F0063277F image!], F [barcode F0063278F image!], F [barcode F0063279F image!], US [barcode US00097302 image!], US [barcode US00997640 image!]).

#### Note.

For the type designation, Cuatrecasas (1941) cited three collections (“*Typus*: … 25-III-1940, *J. Cuatrecasas, 8691*. También en id. id., hondonada del Abra de San Andrés, 2100-1900 m. alt., 24-III-40, *J. Cuatrecasas, 8601 y 8654*”) but he did not mention any herbaria. We chose *J. Cuatrecasas 8691*, conserved at the COL Herbarium, as the lectotype. Bernal (2016: 1128) merely mentioned that the type is at the COL Herbarium and treated this species as a synonym of *W.sorbifolia* Kunth.

### ﻿ Weinmannia sibundoya

Taxon classificationPlantaeOxalidalesCunoniaceae

Cuatrec., Caldasia 2: 23, figs. 10C, 12. 1941.

780D5602-8654-516D-96BB-5E52C0A48303

#### Type.

Colombia • Comisaría del Putumayo, bosque paramero en el filo de la Cordillera en el extremo E. del valle de Sibundoy, La Cabaña, 2800 m. alt., 2-I-1941, *J. Cuatrecasas 11624* (lectotype, designated here, COL [barcode COL000001507!]; isolectotypes, F [barcode F0063280F image!], US [00097305 image!]).

#### Note.

Cuatrecasas (1941) cited two collections as the type (“*Typus*: … 2-I-1941, *J. Cuatrecasas, 11624. También en id. id., no. 11527*.”) and did not mention any herbarium. Neither Bernardi (1961: 165) nor Harling (1999: 22) designated a lectotype. We herewith designate *Cuatrecasas 11624*, conserved at the COL Herbarium, as the lectotype. *Weinmanniasibundoya* is considered a synonym of *W.multijuga* Killip & A.C.Sm. in Harling (ibid.) and Bernal (2016: 1127).

### ﻿ Weinmannia subvelutina

Taxon classificationPlantaeOxalidalesCunoniaceae

Cuatrec., Caldasia 2: 15, figs. 3, 5A. 1941.

0E5DFB5F-2F9C-5CB5-BE4D-7823149E94F3

≡ W.rollottiiKillipvar.subvelutina (Cuatrec.) Bernardi, Candollea 18: 327. 1963.

#### Type.

Colombia • Comisaría del Putumayo, lado sur de la Laguna de la Cocha en La Quebrada de Santa Lucía, 2850 m. alt. 8-1-1941. *J Cuatrecasas 11816* (lectotype, designated here, COL [barcode COL000001513!]; isolectotypes, F [barcode F0063288F!], F [barcode F0063287F!], G [barcode G00357673 image! fragment], US [barcode US00097312 image!]).

#### Note.

Cuatrecasas (1941) cited a single collection as the type but did not mention the herbarium housing the type (“*Typus*: … 8-I-1941, *J. Cuatrecasas, 11816*.”). We herewith designate the COL specimen as the lectotype. We add that Bernardi (1963: 327-328) mentioned “*Cuatrecasas 11816* (COL, F), cum capsulis (typi numerus).” Since he cited two herbaria and the plural term “typi”, his citation does not constitute a typification. *Weinmanniasubvelutina* is considered a synonym of *W.rollottii* Killip in Bernal (2016: 1128).

### ﻿ Weinmannia tamana

Taxon classificationPlantaeOxalidalesCunoniaceae

Cuatrec., Revista Acad. Colomb. Ci. Exact. 5: 32. 1942.

B15F3485-BCAC-55DF-A992-426340DB251D

#### Type.

Colombia • Cordillera Oriental, Departamento de Santander, Páramo de Tamá, vertiente de Samaria, 2600-2900 m. alt., colect. 29 oct. 1941, *J. Cuatrecasas, R. E. Schultes & E. Smith 12726* (lectotype, designated here, COL [barcode COL000001508!]; isolectotypes, F [barcode F0063291F image!], F [barcode F0063292F image!], GH [barcode GH00043362 image!], P [barcode P02441810 image!], U [barcode U0007918 image!], US [barcode US00097313 image!].

#### Note.

Cuatrecasas (1942) cited a single collection as type, but he did not mention in which herbarium the type was housed (“Typus: … *J. Cuatrecasas, R. E. Schultes & E. Smith 12726*.”). Bernardi (1961: 176) cites a duplicate at the F Herbarium, but he does not designate it as a type. We chose the duplicate deposited at the COL Herbarium as the lectotype. Bernal (2016: 3027) treats this species as a synonym of *W.fagaroides* Kunth.

### ﻿ Weinmannia tolimensis

Taxon classificationPlantaeOxalidalesCunoniaceae

Cuatrec., Trab. Mus. Ci. Nat., Ser. Bot. 26: 18, figs 10, 11. 1933.

7BF53AFF-2B02-57B0-8293-904A7CD61472

#### Type.

Colombia • Andes, Cordillera Central ad 3.000 m. alt. in monte Tolima, inter El Salto et La Selva, 16-V-1932, *J. Cuatrecasas 2737* (lectotype, designated here, MA [barcode MA235618!]).

#### Note.

Cuatrecasas (1933) did cite a single collection (“legi [*J. Cuatrecasas*] *no. 2737*.”) but did not use the term type or mention the herbarium housing the collection. We herewith designate the MA Herbarium specimen as the lectotype. *Weinmanniatolimensis* is an accepted species in Bernal (2016: 1128).

### ﻿ Weinmannia tolimensis Cuatrec. var. latifoliolata

Taxon classificationPlantaeOxalidalesCunoniaceae

Cuatrec., Lloydia 11(3): 204. 1948 [1949].

2F8F85D4-962E-564E-8E61-9895F3A2D861

#### Type.

Colombia • Dep. del Cauca; Cordillera Central vertiente occidental Cabeceras del río Palo, Quebrada de Santo Domingo, bosquecillo subiendo al páramo, 2950-3150 m. alt., 13-XII-1944, *J. Cuatrecasas 19256* (lectotype, designated here: F [barcode F0063295F image!]; isolectotypes, F [barcode F0063294F image!], US [barcode US00097315 image!]).

#### Note.

Cuatrecasas (1948 [1949]) cited the single collection *J. Cuatrecasas 19256* housed at the F Herbarium as the type; however, there are two duplicates of this collection at that herbarium, and we chose one of them for the lectotypification (cf. Art. 40 Ex. 3). This variety is a synonym of *W.tolimensis* Cuatrec. according to Bernal (2016: 1128).

##### ﻿Name described by Georg Hieronymus

The following name, included by Harling (1999) in his treatment of Cunoniaceae for Ecuador, is typified here.

### ﻿ Weinmannia cochensis

Taxon classificationPlantaeOxalidalesCunoniaceae

Hieron., Bot. Jahrb. Syst. 21(3): 310. 1895.

D50FE46B-01DE-5B9E-A55C-6383896AC901

#### Type.

Colombia • “*Páramo dicta ad fluvium Rio de Cocha, … in regione suprema silvarum, alt. s. m. 3200 m, mense Augusto fructifera collecta est.*” *A. Stübel 357* (lectotype, designated here, B [barcode B_10_9009851!]).

#### Note.

In the protologue, Hieronymus (1895) cited five Alfons Stübel collections from Colombia, considered as syntypes: “*crescit ad Cuchilla de Santo Domingo inter urbem Popayan et altiplanitiem Páramo de Huila (coll. columb. n. 290*" [B barcode B_10_9009849!, F barcode F0063217F image! fragment]; "*in altiplanitie Páramo dicta ad fluvium Rio de Cocha*, …" (coll. columb. n. *354* [B barcode B_10_9009852!]), "*et haud procul a loco ultimo indicato in regione suprema silvarum, alt. s. m. 3200 m*, …" (coll. columb. n. *357* et *360 d* [B barcode B_10_9009850!]); "*prope lacum Laguna de Pasto alt. s. m. 2800 m*" (coll. columb. n. *365* [B!].” Bernardi (1961: 179) cited two of those collections at Herbarium B (*Stübel 290*, *357*), but did not designate a type, and Harling (1999: 39) cited all five aforementioned collections as syntypes, but he was unable to study any of them. The specimen here chosen as lectotype, *A. Stübel 357*, deposited in the Berlin Herbarium, matches the original description well and has inflorescences; furthermore, the area where it was collected can be easily discerned today based on the label information: “*Excursión de Pasto a la Laguna Grande de Cocha y al Cerro Patascoy (falso): región superior de bosques, 3200 m, viii, 1869.*” The specific epithet refers to ‘Laguna de la Cocha’ in Nariño, Colombia. The other syntypes have rather imprecise label localities, lack inflorescences, or are fragments only. This species is accepted in Bernal (2016: 1126) and Harling (1999: 22).

## Supplementary Material

XML Treatment for Weinmannia balbisiana Kunth var. subovata

XML Treatment for Weinmannia bogotensis

XML Treatment for Weinmannia caquetana

XML Treatment for Weinmannia crenata C. Presl var. caliana

XML Treatment for Weinmannia cundinamarcensis

XML Treatment for Weinmannia duquei

XML Treatment for Weinmannia magnifolia

XML Treatment for Weinmannia myrtifolia

XML Treatment for Weinmannia ovalis Ruiz & Pav. var. petiolata

XML Treatment for Weinmannia parvifoliolata

XML Treatment for Weinmannia penicillata

XML Treatment for Weinmannia queremalensis

XML Treatment for Weinmannia sclerophylla

XML Treatment for Weinmannia sibundoya

XML Treatment for Weinmannia subvelutina

XML Treatment for Weinmannia tamana

XML Treatment for Weinmannia tolimensis

XML Treatment for Weinmannia tolimensis Cuatrec. var. latifoliolata

XML Treatment for Weinmannia cochensis

## ﻿Additional information

### Author ORCIDs

Carmen Ulloa Ulloa https://orcid.org/0000-0003-2453-8131

Francisco Fajardo-Gutiérrez https://orcid.org/0000-0002-2235-3910

Nora H. Oleas https://orcid.org/0000-0002-1948-4119

### Data availability

All of the data that support the findings of this study are available in the main text.
